# Medical College Notes

**Published:** 1896-07

**Authors:** 


### Medical College Notes.

Dental Department, University of Buffalo.—Dr. R. H. Hofheinz, of Rochester, N. Y., has been appointed assistant professor to the chair of operative dentistry which is occupied by Dr. F. E. Howard, of Buffalo. Dr. F. J. Gieser has been appointed professor of chemistry and metallurgy, Dr. G. A. Himmelsbach, professor of general anatomy, and Dr. A. L. Benedict, professor of physiology.

---

Professor Edwin Klebs has been elected to the chair of pathology in Rush Medical College, Chicago.

This college recently has been recognised by the Examining Board of the Royal College of Physicians and the Royal College of Surgeons of London, England. This recognition entitles its alumni to all the privileges accorded to the graduates of other institutions recognised by that Board.

